# Prevalence, Severity, and Clinical Management of Brain Incidental Findings in Healthy Young Adults: MRi-Share Cross-Sectional Study

**DOI:** 10.3389/fneur.2021.675244

**Published:** 2021-05-20

**Authors:** Aïcha Soumaré, Naka Beguedou, Alexandre Laurent, Bruno Brochet, Constance Bordes, Sandy Mournet, Emmanuel Mellet, Edwige Pereira, Clothilde Pollet, Morgane Lachaize, Marie Mougin, Ami Tsuchida, Hugues Loiseau, Thomas Tourdias, Christophe Tzourio, Bernard Mazoyer, Stéphanie Debette

**Affiliations:** ^1^UMR1219 Bordeaux Population Health Center (Team VINTAGE), INSERM-University of Bordeaux, Bordeaux, France; ^2^Neurofunctional Imaging Group, Institute of Neurodegenerative Disease-UMR5293, University of Bordeaux, Bordeaux, France; ^3^Neurofunctional Imaging Group, Institute of Neurodegenerative Disease-UMR5293, CNRS, Bordeaux, France; ^4^Neurofunctional Imaging Group, Institute of Neurodegenerative Disease-UMR5293, CEA, Bordeaux, France; ^5^Department of Neurology, University Hospital Centre Bordeaux Pellegrin Hospital Group, Bordeaux, France; ^6^UMR1219 Bordeaux Population Health Center (Team HEALTHY), INSERM-University of Bordeaux, Bordeaux, France; ^7^Department of Neurosurgery, University Hospital Centre Bordeaux Pellegrin Hospital Group, Bordeaux, France; ^8^Department of Diagnostic and Therapeutic Radiology and Neuroimaging, University Hospital Centre Bordeaux Pellegrin Hospital Group, Bordeaux, France; ^9^Magendie Neurocenter INSERM-U1215, University of Bordeaux, Bordeaux, France; ^10^Department of Medical Information, University Hospital Centre Bordeaux Pellegrin Hospital Group, Bordeaux, France

**Keywords:** incidental findings, brain MRI, prevalence, young adults, epidemiology, multiple sclerosis, radiologically isolated syndrome

## Abstract

**Background and Objectives:** Young adults represent an increasingly large proportion of healthy volunteers in brain imaging research, but descriptions of incidental findings (IFs) in this age group are scarce. We aimed to assess the prevalence and severity of IFs on brain MRIs of healthy young research participants aged 18–35 years, and to describe the protocol implemented to handle them.

**Methods:** The study population comprised 1,867 participants aged 22.1 ± 2.3 years (72% women) from MRi-Share, the cross-sectional brain MRI substudy of the i-Share student cohort. IFs were flagged during the MRI quality control. We estimated the proportion of participants with IFs [any, requiring medical referral, potentially serious (PSIFs) as defined in the UK biobank]: overall, by type and severity of the final diagnosis, as well as the number of IFs.

**Results:** 78/1,867 participants had at least one IF [4.2%, 95% Confidence Interval (CI) 3.4–5.2%]. IFs requiring medical referral (*n* = 38) were observed in 36/1,867 participants (1.9%, 1.4–2.7%), and represented 47.5% of the 80 IFs initially flagged. Referred IFs were retrospectively classified as PSIFs in 25/1,867 participants (1.3%, 0.9–2.0%), accounting for 68.4% of anomalies referred (26/38). The most common final diagnosis was cysts or ventricular abnormalities in all participants (9/1,867; 0.5%, 0.2–0.9%) and in those with referred IFs (9/36; 25.0%, 13.6–41.3%), while it was multiple sclerosis or radiologically isolated syndrome in participants with PSIFs (5/19; 26.3%, 11.5–49.1%) who represented 0.1% (0.0–0.4%) and 0.2% (0.03–0.5%) of all participants, respectively. Final diagnoses were considered serious in 11/1,867 participants (0.6%, 0.3–1.1%). Among participants with referred IFs, 13.9% (5/36) required active intervention, while 50.0% (18/36) were put on clinical surveillance.

**Conclusions:** In a large brain imaging study of young healthy adults participating in research we observed a non-negligible frequency of IFs. The etiological pattern differed from what has been described in older adults.

## Introduction

The widespread use of advanced brain MRI techniques in clinical research entails increasingly frequent detection of incidental findings (IFs). An IF is “a finding concerning an individual research participant that has potential health or reproductive importance and is discovered in the course of conducting research, but is beyond the aims of the study” (1). While most IFs are not clinically meaningful, some can reflect underlying diseases amenable to treatment or can, in rare instances, be life-threatening (e.g., aneurysms, neoplasms). In such cases, early detection and treatment could be of clinical benefit for the participant. Conversely, detecting and disclosing IFs can entail increased clinical workload and costs, cause psychological or financial distress to participants, and expose them to potentially harmful interventions (1–4). However, specific guidelines on the identification and management of brain IFs in a research setting are currently lacking (5–7).

Young adults (18–35 years) participating in biomedical research represent a particularly challenging group regarding IF discovery. First, although individuals in this age group represent a large proportion of “healthy volunteers” in brain imaging research, descriptions of IFs in this age range are scarce. The very few available studies were based on small or selected samples with a broad definition of IFs, low image resolution, and no assessment of their clinical severity (8, 9). Second, characteristics and severity of IFs could differ by age (10, 11), with young asymptomatic adults experiencing greater consequences with longer term effects than older ones. In this context, a systematic description of the prevalence and clinical relevance of IFs in young adults could provide valuable information to notify research participants in this age range of the likelihood and consequences of IFs.

We sought to assess the prevalence of IFs and their severity in 1,867 young adults aged 18–35 years, participating in the MRi-Share brain imaging substudy of the i-Share student cohort, and to describe the standardized protocol implemented to manage them.

## Materials and Methods

### Study Design and Population

The i-Share (internet-based Students' Health Research Enterprise) project^1^ is an ongoing population-based cohort study of French-speaking students that was launched in 2013 (i) to assess the frequency and impact of various diseases or conditions affecting young adults, and (ii) to explore the pathophysiology and early mechanisms underlying common chronic disorders, including diseases occurring at a later age. To be eligible, students had to be officially registered at a University or another higher education institution (HEI), be at least 18 years of age, and be able to read and understand French (12). Students were informed about the objectives of the study through promotion campaigns (flyers, information booths on admission days, lectures, social media, and newsletters). Overall, the study was conducted in >420 universities or HEIs (96% in France), the largest recruitment coming from the universities of Bordeaux, Versailles-Saint-Quentin-en-Yvelines, and Nice-Sophia-Antipolis.

The MRi-Share ancillary study is a brain imaging study embedded within the i-Share cohort, which entails a brain MRI and a battery of cognitive tests (13). The objectives of MRi-Share are (i) to characterize morphological and functional variability of the brain in young adults by building a database of morphological and functional MRI images, (ii) to describe the anatomical and functional brain architecture in this population, and (iii) to characterize brain connectivity and its relation with cognitive skills. To participate in MRi-Share, i-Share participants had to be aged between 18 and 35 years, to be registered in a University or HEI in the Bordeaux area, to have completed the i-Share baseline self-administered online questionnaire, and signed an informed consent. Participants with a contraindication to brain MRI (e.g., claustrophobia, pacemaker, and other implanted electronic or metal devices), pregnancy or nursing were not eligible. Between October 2015 and June 2017, i-Share participants were invited to take part in MRi-Share. Those interested were then invited for a visit during which they received detailed information on this ancillary study and were given a virtual tour of the MRI facility. Contraindications for the brain MRI were verified and a referent physician answered any questions they had before signing a written informed consent. MRi-Share participants received a compensation of 40 euros.

### MRI Protocol

The MRI acquisition protocol for MRi-Share (13) was designed to emulate that of the UK biobank (UKB) MR imaging study (14) as much as possible, to enable combined analyses of the two databases, since early adulthood is currently not covered by the UKB design. MRIs were performed on a Siemens 3T Prisma scanner (Erlangen, Germany) with a 64-channel head coil (gradients: 80 mT/m−200 T/m/s), between November 2015 and November 2017 at the Bordeaux Institute of BIOimaging. The acquisition lasted about 45 min and included the following five sequences: T1-weighted (T1w) structural imaging (3D MPRAGE, sagittal acquisition, TR/TE/TI = 2,000/2.0/880 ms, repeat × 2, 1 mm^3^ isotropic, 192 × 256 × 256); T2-weighted (T2w) FLAIR structural imaging (3D SPACE, sagittal acquisition, TR/TE/TI = 5,000/394/1800 ms, repeat × 2, 1 mm^3^ isotropic, 192 × 256 × 256); Diffusion Weighted Imaging [DWI, axial acquisition, echoplanar imaging, TR/TE = 3,540/75.0 ms, multiband × 3, 100 directions, multishell b = 0 s/mm^2^ (8 + 8 phase-encoding reversed), b = 300 s/mm^2^ (8 directions), b = 1,000 s/mm^2^ (32 directions), b = 2,000 s/mm^2^ (60 directions), 1.75 mm^3^ isotropic, 118 × 118 × 84]; Susceptibility-Weighted Structural Imaging (SWI, axial acquisition, TR/TE1 = 24.0/9.42 ms, 0.8 × 0.8 × 3 mm^3^ anisotropic, 252 × 288 × 48); and Resting-state functional MRI (2D T2^*^-BOLD resting state, axial acquisition, echoplanar imaging, TR/TE = 850/35.0 ms, multiband × 6, 2.4 mm^3^ isotropic, 88 × 88 × 66). A detailed summary of acquisition parameters for each modality is presented in Supplementary Table 1. Following the MRI scan, each participant had to complete a questionnaire about their thoughts while undergoing the functional MRI and to perform two cognitive tests during 20 min.

### Protocol for Assessment and Management of Incidental Findings

#### Definitions and Assessment of IFs

This study is focused on IFs identified on structural brain MRI exclusively. Within days following the MRI acquisition, T1w and T2w FLAIR images were systematically checked visually for quality by one of two MD investigators trained in brain imaging with >30 years' experience [EM, BM (also professor of neuroradiology at Bordeaux University Hospital)]. If an IF (defined as proposed previously) (1) was detected during this quality control and considered to be potentially harmful for the participant's health, it was shown to a specialized clinical neuroradiologist at Bordeaux University Hospital (TT, professor of neuroradiology with >12 years' experience) who checked the clinical relevance of this IF to decide whether it required medical referral. DWI and/or SWI images were used to better characterize IFs detected on T1w and T2w FLAIR. However, raw DWI and/or SWI images did not undergo visual quality control because those modalities are prone to artifacts induced by eddy currents and/or susceptibility effects; efficient quality control of these acquisitions must be performed after some pre-processing as described previously (13). Of note, the following IFs were not reported: (i) T2-hyperintensities that were isolated or in small numbers (<5), and without any features suggestive of an underlying inflammatory condition such as ovoid shape and periventricular, juxtacortical, or posterior fossa location; (ii) small pineal cysts. For the latter, in the absence of recommendations the threshold was initially set at 10 mm (until May 2016), and subsequently at 15 mm, as the 10 mm threshold generated too many cases and a size >15 mm was described to be potentially associated with neurologic symptoms attributable to mass effect on adjacent structures or hydrocephalus through the compression of the cerebral aqueduct (15). All IFs flagged as requiring referral were reported to a referent neurologist at Bordeaux University Hospital (SD), and categorized as requiring immediate (e.g., acute stroke, encephalitis), urgent (within 1 week, e.g., malignant brain tumor), or routine medical referral. Prior to consenting to participate, MRi-Share participants were informed beforehand that the study might, in rare instances, entail the discovery of an IF, for which they might be contacted if considered to be potentially harmful for their health, with detailed information provided in the setting of a medical visit by a certified neurologist. i-Share participants volunteering for MRi-Share who refused to receive feedback about a potential IF were not eligible for MRi-Share. Participants were also informed that the MRI exam was not a diagnostic test and that some anomalies might not be detected.

#### Disclosure and Handling of Referred IFs

The referent neurologist called the participant <48 h before the next available neurological outpatient clinic slot, in order to minimize the period of anxiety and stress. No diagnosis was given by phone; participants were informed of the presence of an imaging finding on their MRI scan requiring additional investigation and an appointment in the outpatient clinic was organized. In the outpatient clinic, the neurologist explained the observed abnormality to the participant, collected information about medical history and ongoing treatments, conducted a physical examination, and informed the participant about the proposed follow-up procedures, i.e., additional imaging, blood tests and/or referral to another physician (if needed for diagnosis or management purposes, as described in the letter of information received prior to providing consent). Psychological support was also proposed at the Student Health Service center when needed. Following this interview with the referent neurologist, the participant was systematically invited to undergo a complementary “clinical” brain imaging (MRI or CT-scan, with or without contrast enhancement depending on the nature of the IF), which was interpreted by a clinical neuroradiologist in the context of clinical care. Once the final diagnosis was confirmed, the appropriate care was determined by a specialist on the basis of the type, location, severity, size, and progression of the IF, as well as clinical symptoms and medical history.

#### Potentially Serious Incidental Findings (PSIFs) and Severity of Final Diagnosis

In order to facilitate the comparison with published studies, we retrospectively classified IFs requiring medical referral as potentially serious incidental findings (PSIFs) using a protocol developed by the UKB that was not available at the time of our study (11, 16, 17). First, IFs were defined as PSIFs if listed as such by the UKB or if meeting their definition of PSIF, i.e., an IF “indicating the possibility of a condition which, if confirmed, would carry a real prospect of seriously threatening lifespan, or of having a substantial effect on major body functions or quality of life” (17). Finally, for participants with PSIFs who were followed-up, final diagnoses were considered as “either: serious (if they were likely to threaten lifespan, or have a substantial impact on quality of life or major body function); not serious (if this was not the case or if the diagnosis was already known); or indeterminate (if there remained insufficient data to classify a final diagnosis as serious or not)” (11). In participants with more than one PSIF, the most serious final clinical diagnosis was accounted for.

### Other Measurements

Information about sociodemographic and academic characteristics, health status, personal and family medical history, and lifestyle habits were collected through the i-Share baseline self-administered online questionnaire. The following variables were considered in the analyses to describe the study sample: age at i-Share inclusion, age at MRI, sex, field of study (healthcare/health related disciplines vs. others), self-rated health (defined on a qualitative scale as very good or good vs. fair, bad or very bad), having visited a physician in the past 12 months, regular consumption of medications, history of hospitalization in the past 12 months, familial economic situation during childhood (rated as very comfortable or comfortable vs. fair, difficult or very difficult), current sources of income (familial, scholarship on social grounds, and income-generating activities during the University year), self-reported physician-diagnosed migraine, self-reported physician-diagnosed type 1 diabetes, self-reported physician-diagnosed multiple sclerosis (MS), family history of stroke or cardiovascular disease (myocardial infarction, angina pectoris), family history of cancer, being a current smoker, heavy drinking habits in the past 12 months [defined as frequent episodes of binge drinking (≥6 drinks in about 2 h on the same occasion) 2–6 times per week or every day], use of psychoactive drugs in the past 12 months (cannabis, ecstasy/3,4 methylenedioxymethamphetamine, amphetamines, nitrous oxide, inhalant, or cocaine), and use of other illicit drugs at least once in a lifetime (magic mushrooms or other hallucinogenic plants, crack/free-base, heroin, LSD, or ketamine).

### Statistical Analyses

To assess whether MRi-Share participants were representative of i-Share participants at large, we compared characteristics of i-Share participants recruited at universities or other HEIs in the Bordeaux area with respect to their participation in MRi-Share. We first conducted univariate analyses (analysis of covariance for continuous variables, chi-square or Fisher exact test for categorical variables). Second, we performed multivariable logistic regression including all variables associated with MRi-Share participation in univariate analyses with a *p* ≤ 0.05 in the model. Third, we compared MRi-Share participants' characteristics according to the presence or absence of IFs. Finally, we extracted data from radiological and medical reports to give descriptive statistics: number and proportion of participants with IFs (any, referred, PSIFs) with their corresponding 95% Confidence Intervals (CI) when appropriate: overall, by type of diagnosis and severity; number of IFs. We also presented the management of IFs. All analyses were performed using SAS software version 9.4 (SAS Institute Inc., Cary, NC, USA), and a two-tailed *p* ≤ 0.05 was considered statistically significant.

## Results

**Figure 1:**
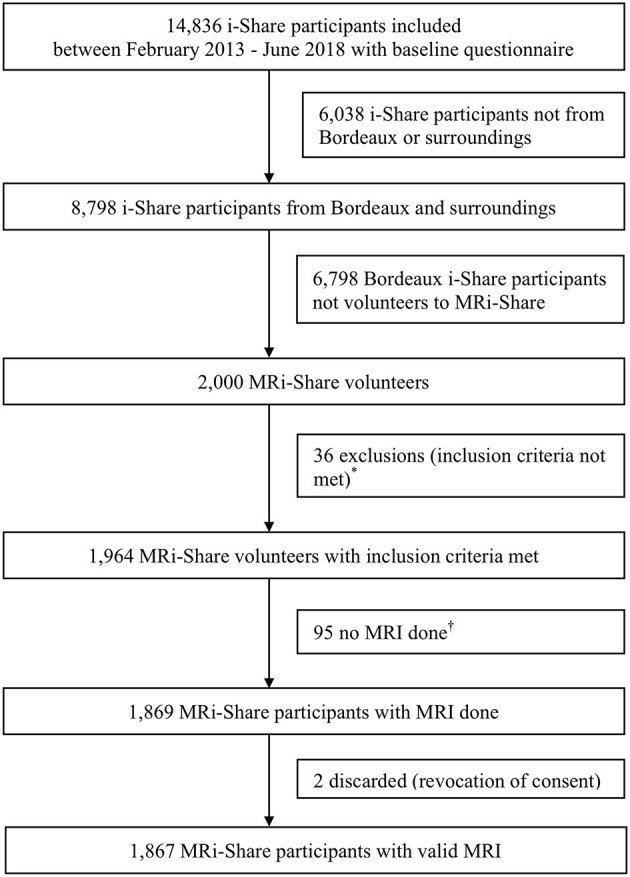
MRi-Share flow diagram. *Age criteria not met, *n* = 4; baseline questionnaire not completed, *n* = 1; presence of contra-indications to MRI, *n* = 16 (metal fragments or devices, *n* = 3; claustrophobia, *n* = 4; others, *n* = 9); others, *n* = 15. ^†^Drop-out, *n* = 54; study termination, *n* = 41.

Of the 14,836 students included in i-Share with a completed baseline questionnaire at the end of the inclusion period for the MRi-Share ancillary study, 8,798 were recruited in universities or other HEIs in Bordeaux or surroundings. Of these, 2,000 students responded to the MRi-Share invitation with 1,964 meeting inclusion criteria. After excluding 95 participants for whom the MRI could not be performed (drop out or termination of study participation) and two participants who rescinded their consent, our final MRi-Share study sample comprised 1,867 participants (Figure 1). Baseline characteristics of the 1,867 students included in MRi-Share are presented in Table 1. Our study population comprised 72% of women with a mean age (SD) at i-Share inclusion and at MRI of 21.2 (2.3) years and 22.1 (2.3) years, respectively. Among i-Share participants, participation in the MRi-Share study was associated with older age, healthcare or health related studies, having had a comfortable or very comfortable familial economic situation in childhood, having an income originating from the family or having a scholarship on social grounds, and use of psychoactive drugs. Migraine and current smoking were less common in MRi-Share participants than in i-Share participants who did not take part in the brain imaging study (multivariable logistic regression analysis, Table 1).

**Table 1:** Comparison of MRi-Share participants with other participants of i-Share Bordeaux.

	MRi-Share participants with MRI^*^ (*N* = 1,867)	**Other Bordeaux i-Share participants** **(*N* = 6,798)**	***P*^†^**	**OR (95% CI)^‡^**	***P*^‡^**
**Age at MRI, years, mean (SD)**	22.1 (2.3)				
**Age at i-Share recruitment, years, mean (SD)**	21.2 (2.3)	20.7 (2.8)	<0.001	1.07 (1.05–1.09)	<0.001
**Male Gender**	27.8 (519)	24.2 (1,648)	0.002	1.10 (0.97–1.24)	0.14
**Field of study**					
Other	46.2 (861)	71.3 (4,845)	<0.001	1.00	<0.001
Healthcare	53.8 (1,002)	28.7 (1,953)		2.68 (2.41–3.00)	
**Self-rated health**					
Fair, bad or very bad	17.5 (327)	20.2 (1,371)	0.01	1.00	0.47
Very good or good	82.5 (1,539)	79.8 (5,427)		1.05 (0.92–1.21)	
**PCP visits 12 months before i-Share inclusion**	87.1 (1,626)	86.1 (5,850)	0.23		
**Regular medication**	23.9 (446)	23.1 (1,567)	0.44		
**Hospitalization 12 months before i-Share inclusion**	16.7 (312)	17.5 (1,193)	0.40		
**Familial economic situation during childhood**					
Fair, difficult or very difficult	42.0 (783)	49.6 (3,371)	<0.001	1.00	0.002
Very comfortable or comfortable	58.0 (1,083)	50.4 (3,427)		1.21 (1.07–1.36)	
**Source of income**					
Family	81.8 (1,528)	79.6 (5,412)	0.03	1.20 (1.03–1.39)	0.02
Scholarship on social grounds	37.9 (707)	42.6 (2,899)	0.001	1.16 (1.02–1.31)	0.02
Activities during University year	41.0 (766)	37.0 (2,517)	0.002	1.11 (0.99–1.24)	0.08
**Self-reported physician-diagnosed migraine**	18.3 (342)	22.9 (1,560)	<0.001	0.85 (0.75–0.98)	0.02
**Self-reported physician-diagnosed type 1 diabetes**	0.4 (8)	0.4 (24)	0.63		
**Self-reported physician-diagnosed MS**	0.1 (1)	0.1 (7)	0.99		
**Family history of CVD or stroke**	14.5 (248)	13.8 (843)	0.45		
**Family history of cancer**	11.5 (203)	11.4 (726)	0.87		
**Current smoker**	29.1 (543)	32.1 (2,181)	0.01	0.81 (0.72–0.93)	0.002
**Heavy drinking 12 months before i-Share inclusion**	5.1 (92)	4.1 (268)	0.07		
**Use of psychoactive drugs 12 months before i-Share inclusion^§^**	47.4 (873)	39.4 (2,623)	<0.001	1.44 (1.27–1.62)	<0.001
**Use of other illicit drugs at least once in a lifetime^¶^**	3.9 (73)	4.3 (289)	0.49		

*Data were collected through the baseline i-share questionnaire before the MRI was done. Values are percentages (number) unless stated otherwise. PCP, Primary care physician; MS, Multiple Sclerosis; CVD, cardiovascular disease*.

*Heavy drinking in the past 12 months is defined as frequent episodes of binge drinking (≥six drinks in about 2 h on the same occasion) 2–6 times per week or every day*.

* *Bordeaux i-Share participants included in MRi-Share ancillary study (fulfilling inclusion criteria, with a signed consent), and a valid MRI*.

† *Analysis of covariance for continuous variables; chi-square or Fisher exact test for categorical variables*.

‡ *Odds ratios (OR) and p-values obtained from Multivariable logistic regression models including all variables significantly associated with the participation in MRi-Share in univariate analyses (with a p ≤ 0.05 in analyses)*.

§ *Cannabis, ecstasy/3, 4-Methylenedioxymethamphetamine, amphetamines, nitrous oxide, inhalant, or cocaine*.

¶ *Magic mushrooms or other hallucinogenic plants, crack/free-base, heroin, LSD, or ketamine*.

IFs were detected in 78 of the 1,867 participants (4.2%, 95% CI: 3.4–5.2%). IFs requiring medical referral (*n* = 38) were found in 36 of those 1,867 participants (1.9%, 1.4–2.7%), two participants having two IFs. Referral was deemed urgent for one participant (0.05%, 0.0–0.3% of the study sample), and routine for the others. Twenty six (68.4%) of the 38 IFs referred were retrospectively characterized as PSIFs [in 25/1,867 individuals (1.3%, 0.9–2.0%)]. Indeed, some lesions that in our opinion required medical referral were not considered as such in the UKB; moreover, for some lesions that were most likely not serious, we preferred to formally rule out differential diagnoses or rare complications by conducting follow-up investigations (e.g., brain MRI with contrast enhancement, electroencephalography). Participants with IFs (any, requiring medical referral, or PSIFs) did not significantly differ from those without in terms of baseline characteristics (Table 2).

**Table 2:** Baseline characteristics of MRi-Share participants with MRI according to incidental findings (IFs) status^*^.

	**Participants without IFs**	**Participants with any IFs**	**Participants with IFs referred**	**Participants with PSIFs^†^**	***P*^‡^**	***P*^§^**	***P*^¶^**
	**(*N* = 1,789)**	**(*N* = 78)**	**(*N* = 36)**	**(*N* = 25)**			
**Age at MRI, years, mean (SD)**	22.1 (2.3)	22.2 (2.5)	22.4 (2.6)	22.5 (2.2)	0.79	0.40	0.33
**Age at i-Share recruitment, years, mean (SD)**	21.2 (2.3)	21.3 (2.7)	21.5 (2.8)	21.8 (2.3)	0.75	0.43	0.23
**Complementary brain imaging, median (min, max)**			1.0 (0.0–4.0)	1.0 (0.0–4.0)			
**Visits with medical specialists, median (min, max)**			1.0 (0.0–6.0)	2.0 (0.0–6.0)			
**Male Gender**	27.6 (493)	33.3 (26)	22.2 (8)	20.0 (5)	0.27	0.48	0.40
**Field of study**							
Other	46.3 (827)	43.6 (34)	50.0 (18)	44.0 (11)	0.63	0.66	0.82
Healthcare	53.7 (958)	56.4 (44)	50.0 (18)	56.0 (14)			
**Self-rated health**							
Fair, bad or very bad	17.5 (313)	17.9 (14)	27.8 (10)	28.0 (7)	0.92	0.11	0.18
Very good or good	82.5 (1,475)	82.1 (64)	72.2 (26)	72.0 (18)			
**PCP visits 12 months before i-Share inclusion**	87.1 (1,558)	87.2 (68)	88.9 (32)	88.0 (22)	0.99	0.76	0.99
**Regular medication**	24.1 (431)	19.2 (15)	30.6 (11)	36.0 (9)	0.32	0.37	0.17
**Hospitalization 12 months before i-Share inclusion**	16.5 (295)	21.8 (17)	27.8 (10)	28.0 (7)	0.22	0.07	0.17
**Familial economic situation during childhood**							
Fair, difficult or very difficult	41.7 (746)	47.4 (37)	50.0 (18)	48.0 (12)	0.32	0.32	0.53
Very comfortable or comfortable	58.3 (1,042)	52.6 (41)	50.0 (18)	52.0 (13)			
**Source of income**							
Family	81.8 (1,464)	82.1 (64)	77.8 (28)	76.0 (19)	0.96	0.53	0.44
Scholarship on social ground	37.6 (672)	44.9 (35)	47.2 (17)	56.0 (14)	0.19	0.24	0.06
Activities during University year	41.3 (739)	34.6 (27)	38.9 (14)	36.0 (9)	0.24	0.77	0.59
**Self-reported physician-diagnosed migraine**	18.2 (325)	21.8 (17)	25.0 (9)	28.0 (7)	0.42	0.29	0.20
**Self-reported physician-diagnosed type 1 diabetes**	0.4 (7)	1.3 (1)	2.8 (1)	4.0 (1)	0.29	0.15	0.11
**Self-reported physician-diagnosed MS**	0.1 (1)	0.0 (0)	0.0 (0)	0.0 (0)	0.99	0.99	0.91
**Family history of CVD or stroke**	14.8 (243)	7.2 (5)	9.7 (3)	15.0 (3)	0.08	0.61	0.99
**Family history of cancer**	11.3 (191)	16.7 (12)	15.2 (5)	22.7 (5)	0.17	0.42	0.10
**Current smoker**	29.5 (527)	20.5 (16)	22.2 (8)	16.0 (4)	0.09	0.34	0.14
**Heavy drinking 12 months before i-Share inclusion**	5.1 (88)	5.6 (4)	9.1 (3)	4.3 (1)	0.78	0.24	0.99
**Use of psychoactive drugs 12 months before i-Share inclusion^||^**	47.7 (841)	41.6 (32)	42.9 (15)	37.5 (9)	0.29	0.57	0.32
Use of other illicit drugs at least once in a lifetime^**^	4.0 (72)	1.3 (1)	2.8 (1)	4.0 (1)	0.37	0.99	0.99

*Data were collected through the baseline i-share questionnaire before the MRI was done. Values are percentages (number) unless stated otherwise. PCP, Primary care physician; MS, Multiple Sclerosis; CVD, cardiovascular disease*.

*Heavy drinking in the past 12 months is defined as frequent episodes of binge drinking (≥six drinks in about 2 h on the same occasion) 2–6 times per week or every day*.

* *Bordeaux i-Share participants included in MRi-Share ancillary study (fulfilling inclusion criteria, with a signed consent) and a valid MRI (N = 1,867)*.

† *PSIFs (Potentially Serious Incidental Findings) are IFs referred that were retrospectively identified according to the list of PSIFs developed by the UK biobank, or its definition of PSIFs*.

‡ *Analysis of covariance for continuous variables or chi-square/Fisher exact test for categorical variables are used to compare: participants without IFs vs. those with IFs*;

§ *participants without IFs vs. those with IFs referred*;

¶ *participants without IFs vs. those with PSIFs*.

|| *Cannabis, ecstasy/3, 4-Methylenedioxymethamphetamine, amphetamines, nitrous oxide, inhalant, or cocaine*.

** *Magic mushrooms or other hallucinogenic plants, crack/free-base, heroin, LSD, or ketamine*.

**Figure 2:**
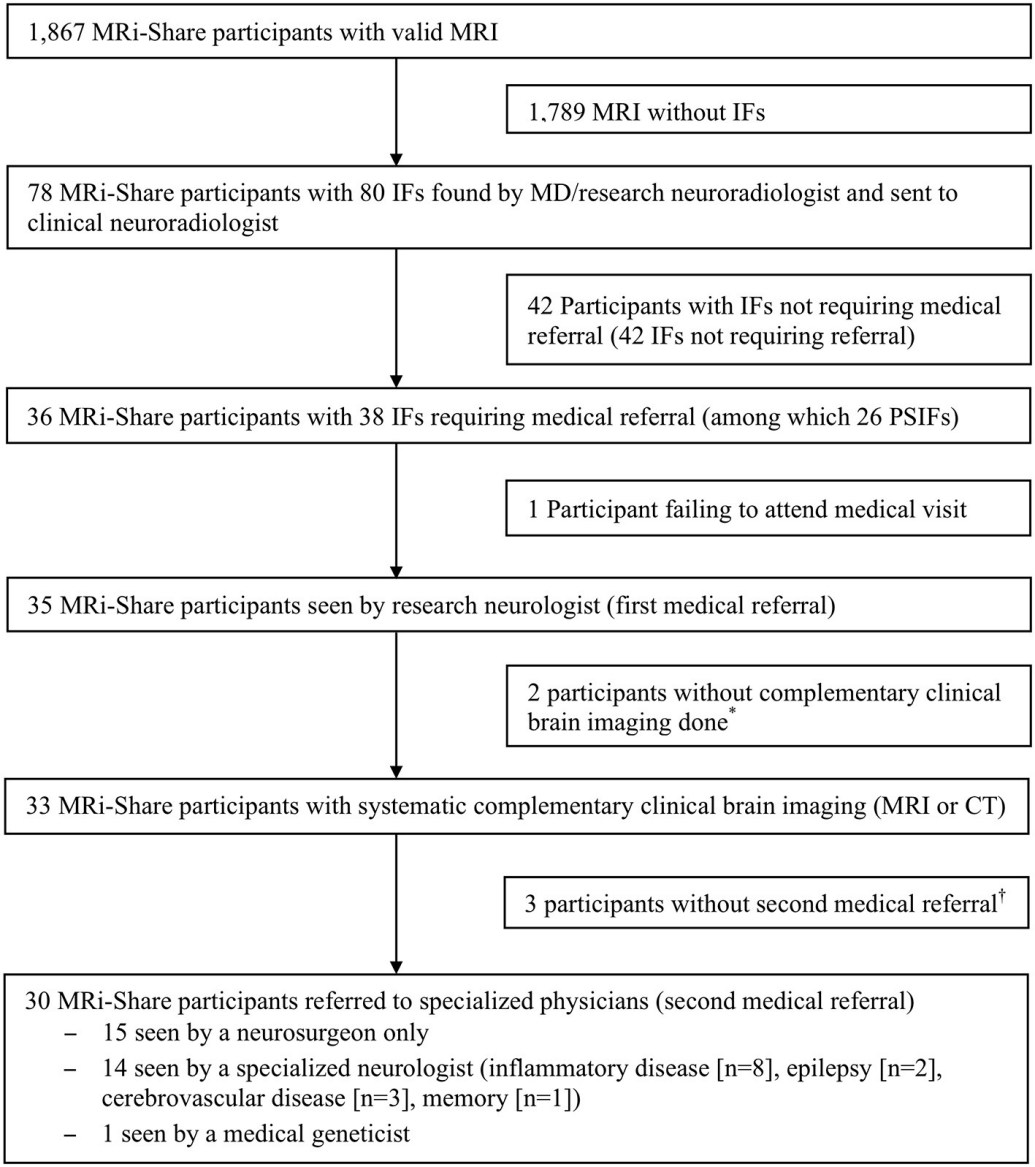
Procedure for detection and management of incidental findings (IFs). MD, medical doctor; PSIFs, potentially serious incidental findings. *Refusal by the participant, *n* = 1; not necessary (previous reassuring MRI performed prior to i-Share research MRI), *n* = 1. ^†^Refusal of follow-up visit, *n* = 2; not necessary, *n* = 1.

The procedure for detection and management of IFs is outlined in Figure 2. Of the 36 participants with IFs requiring medical referral, 35 (97.2%) were seen by the referent neurologist, and 33 (91.7%) underwent the recommended clinical brain imaging (MRI or CT). One participant remained unreachable after failing to attend the scheduled appointment with the referent neurologist and another participant refused the recommended clinical brain imaging. In both cases, the primary care physician was informed about the IF, with participants' prior consent. In one participant, the IF was retrospectively found to have already been diagnosed on a previous clinical brain MRI prior to MRi-Share participation. In total, 30 out of the 33 (90.9%) participants with a complementary brain imaging were seen by at least one additional medical specialist.

Table 3 presents the initial putative diagnosis for the 38 IFs of the 36 participants requiring medical referral. After additional medical referral and ancillary examinations, the suspected diagnosis changed for five of the 36 participants referred (13.8%, Table 3). No conclusion could be drawn on the final diagnosis for three participants, i.e., the one who failed to attend the visit with the referent neurologist, the one who refused the complementary clinical imaging, and one who underwent the complementary brain MRI but did not attend additional visits and ancillary examinations requested for an accurate diagnosis. All participants with an IF seen by the referent neurologist underwent a thorough clinical interview to rule out any prior symptoms that could retrospectively be attributed to the IF. Some were prescribed additional ancillary investigations, such as an electroencephalography if the IF was deemed to be a potential source of epilepsy. In participants with T2 abnormalities suggestive of demyelination based on their location, size, and morphology, a brain and spinal cord MRI with gadolinium injection and a lumbar puncture were performed.

**Table 3:** Diagnosis' classification of the 38 incidental findings (IFs) of the 36 participants requiring medical referral according to etiology.

**Initial diagnosis on research MRI**		***N***	**Final diagnosis after clinical brain imaging and assessment**		***N***
**(*****N*** **=** **36 participants)**			**(*****N*** **=** **33 participants)**		
**Cysts/ventricular abn**. (*n* = 9)	Pineal cyst	5	**Cysts/ventricular abn**. (*n* = 9)	Pineal cyst	5
	**Ventriculomegaly with stenosis of aqueduct of Sylvius^‡^**	1		**Passive hydrocephalus with stenosis of aqueduct of Sylvius^‡^^§^**	1
	**Arachnoid cyst^†^**	3		**Arachnoid cyst^†^^§^**	2
				**Arachnoid cyst^†^**	1
**Vascular anomaly** (*n* = 7)	**Cavernoma^‡^**	4	**Vascular anomaly** (*n* = 6)	**Cavernoma^‡^^§^**	4
	DVA	2		DVA	2
	dPVS^*^^†^	1			
**Inflammatory WMH** (*n* = 12)	RIS or MS^*^^‡^	11	**Inflammatory WMH** (*n* = 5)	**MS^‡^^§^**	2
	Inflammatory leukoencephalopathy^*^^‡^	1		**RIS^‡^^¶^**	3
**Tumors** (*n* = 3)	**Ependymoma^†^**	1	**Tumors** (*n* = 4)	**Ependymoma^†^^§^**	1
	**MVNT^†^**	2		**MVNT^†^^¶^**	2
				**Ganglioglioma^†^^§^**	1
**Cortical malformations** (*n* = 3)	Neuronal migration disorder	2	**Cortical malformations** (*n* = 3)	Neuronal migration disorder	2
	**Focal cortical dysplasia^‡^**	1		**Focal cortical dysplasia^‡^^¶^**	1
**Other, neurological** (*n* = 1)	**Basal ganglia calcifications^‡^**	1	**Other, neurological** (*n* = 5)	**Fahr's syndrome^‡^^¶^**	1
				WMH without underlying inflammatory disease	3
				Undetermined leukoencephalopathy	1
Other, non-neurological (*n* = 3)	Hypertrophy lymphoid tissue in cavum	1	Other, non-neurological (*n* = 3)	Hypertrophy lymphoid tissue in cavum	1
	Bone lesion	1		Benign bone lesion	1
	Lesion or retention in sinus	1		Cyst in sinus	1

*abn., abnormalities; DVA, Developmental Venous Anomaly; MVNT, Multinodular and Vacuolating Neuronal Tumor; dPVS, dilated Virchow Robin Space; WMH, White Matter Hyperintensities; RIS, Radiologically Isolated Syndrome; MS, Multiple Sclerosis. Thirty-four participants referred have one IF; two participants have two IFs*.

*Initial diagnosis could not be confirmed for three inflammatory WMH suggestive of RIS or MS*.

* *Initial diagnosis modified after additional medical referral and ancillary examinations: one dPVS (vascular anomaly) turned out to be a ganglioglioma (tumor), three inflammatory WMH suggestive of RIS or MS turned out to be WMH without underlying inflammatory disease, one inflammatory WMH suggestive of inflammatory leukoencephalopathy turned out to be an undetermined leukoencephalopathy*.

*In bold, Potentially Serious Incidental Findings (PSIFs) are IFs referred that were retrospectively identified according to*:

† *the list of PSIFs developed by the UK biobank, or*

‡ *the UK biobank definition of PSIFs*.

*Final diagnosis classified as*

§ *serious, or*

¶ *indeterminate, according to the UK biobank definition of diagnosis severity in participants with PSIFs followed-up*.

Cysts/ventricular abnormalities were the most frequent referred IFs in MRi-Share, detected in nine of 1,867 participants (0.5%, 95% CI: 0.2–0.9% of the study sample), comprising pineal cysts, arachnoid cysts, and hydrocephalus (Table 3, Supplementary Table 2). Vascular abnormalities, composed primarily of cavernomas, were the second most common referred IFs in the study sample, observed in six participants (0.3%, 0.1–0.7%). They were followed by white matter hyperintensities suggestive of inflammatory disease observed in five participants (0.3%, 0.09–0.7%), of whom two were diagnosed with MS (0.1%, 0.0–0.4%) according to the McDonald criteria (18), and three with Radiologically Isolated Syndrome (RIS) (0.2%, 0.03–0.5%) based on the Okuda and DIS-Barkhof criteria (19, 20). One of the two MS patients had initially been diagnosed with a RIS. Other IFs included cortical malformations, non-neurological IFs, and Fahr's syndrome (Table 3, Supplementary Table 2).

Among participants with PSIFs according to the UKB list or definition and follow-up available for a final diagnosis (*n* = 19), RIS or MS were the most common IFs (26.3%, Supplementary Table 2). Serious diagnoses, as defined in the UKB (11), occurred in 11/1,867 participants (0.6%, 0.3–1.1% of total study sample), representing 57.9% (36.2–76.9%) of participants with PSIFs and a final diagnosis (11/19) (Table 3). Conversely, non-serious and indeterminate diagnoses occurred in one (0.05%, 0.0–0.3% of the study sample) and seven (0.4%, 0.2–0.8% of the study sample) participants, respectively.

Regarding the management of identified IFs, active intervention was required for five participants with referred IFs (also defined as PSIFs) (0.3%, 0.09–0.7% of the study sample; 13.9%, 5.6–29.1% of participants with referred IFs), and comprised surgery, medical treatment, or both. Clinical surveillance with or without follow-up brain imaging was prescribed for 18 participants with referred IFs (1.0%, 0.6–1.5% of the study sample; 50.0%, 34.5–65.5% of participants with referred IFs).

## Discussion

In 1,867 young students (aged 18–35 years) who underwent 3T brain MRI as part of their participation in the MRi-Share research project, IFs were detected overall in 4.2% (3.4–5.2%) of participants, and IFs requiring medical referral in 1.9% (1.4–2.7%) of participants. The frequency of PSIFs according to the UKB list or definition was 1.3% (0.9–2.0%), while final diagnoses were considered serious in 0.6% (0.3–1.1%) of the participants. The leading final diagnosis was cysts or ventricular abnormalities in participants with referred IFs (25.0%), while it was MS or RIS among those with PSIFs followed-up (26.3%). In this young student population, the prevalence of MS and RIS was, respectively, 0.1% (0.0–0.4%) and 0.2% (0.03–0.5%).

### Comparison With Other Studies and Implications of Findings

To our knowledge, only two studies have focused on brain IFs specifically in healthy young adults (8, 9), describing a prevalence of IFs between 5.8 and 9.4%. In one study (*N* = 2,536 men, mean age: 20.5 years) (8), IFs were defined as abnormal findings based on the sole judgment of one general radiologist. In the other study (*N* = 203; mean age: 21.9 years) (9), scans were read by a neuroradiologist, but IFs could also broadly include variations of the norm. A subsequent meta-analysis suggested that the rate of PSIFs in these two studies was in fact lower than in MRi-Share, between 0.5 and 0.7% (17) vs. 1.3%. Potential explanations for these differences with MRi-Share findings are several fold, including differences in sample characteristics, MRI methodology (1–1.5T vs. 3T MRI and advanced sequences such as SWI in MRi-Share), and IF definitions. Differences in detection protocols [single reader—in one case non-specialty (8) vs. two consecutive subspecialty readers with >12–30 years' experience in brain imaging in MRi-Share] can also be highlighted. Indeed, lesion detection in neuroradiology settings relies on the level of expertise and experience of the reader, with non-specialty readers (non-neuroradiologists) performing at lower accuracy compared with subspecialty ones (neuroradiologists) (21); moreover, blind double interpretation is supposed to reduce diagnostic errors in radiology particularly with the added value of a specialist neuroradiology second opinion vs. a general radiologist (22). Recently, the UKB assessed the prevalence, type, and final clinical diagnosis of PSIFs in 7,334 middle-aged and older research participants (40–69 years; median age: 63 years) (11). MRI machines (Siemens Prisma) and acquisition parameters were the same as in MRi-Share by design (13). In the UKB, brain PSIFs were detected in 58 participants (0.8%, 0.6–1.0%) using two protocols: a systematic review by a radiologist for the first 1,000 scans (protocol 1, 2.3%), and a radiographer flagging for confirmation by a radiologist for the subsequent 6,334 scans (protocol 2, 0.6%). Serious diagnoses occurred in 0.2% (0.1–0.4%) of the sample (protocol 1, 0.4%; protocol 2, 0.2%) representing 29.3% of those with PSIFs. The slightly higher prevalence of PSIFs and serious diagnoses in MRi-Share could be due to differences in the age of participants (although more IFs would be expected with increasing age) (23), selection bias, and systematic reading by a neuroradiologist or MD investigators highly trained in brain imaging and subsequently by a highly trained clinical neuroradiologist in MRi-Share. Finally, a systematic review and meta-analysis of 16 neuroimaging studies (*N* = 19,559 individuals) (24) and an umbrella review of two systematic reviews (*N* = 27,316 individuals) (25) reported IF discovery rates of 2.7 and 22%, respectively. However, these two studies did not provide any additional results for young adults beyond the aforementioned studies (8, 9).

While cysts and vascular anomalies were the most common brain IFs overall in MRi-Share (a quarter of referred IFs), consistent with prior research in young and pediatric populations (8, 9, 26), PSIFs and serious IFs were both dominated by MS or RIS (nearly a quarter of cases). MS and RIS were not described in middle-aged and older adults from UKB, where tumors were the dominating PSIFs, mostly of a different type (meningioma, pituitary tumor, vestibular schwannoma) than the few tumors seen in MRi-Share (ependymoma, multinodular and vacuolating neuronal tumor). A similar variation of tumor histological subtypes by age was recently reported by the Central Nervous System tumor registry of the Bordeaux (Gironde) region in France (27).

The relatively high frequency of MS and RIS in this sample of healthy young adults (0.3% in total, of which 0.2% for RIS) has important implications. MS is a potentially disabling neurological disease with a considerable impact on quality of life (28). There is converging evidence that patients with MS and early initiation of disease modifying therapy (DMT) have a more favorable outcome with a lower frequency of clinical attacks. RIS is a syndrome described for the first time in 2009 and defined by incident MRI findings typical of MS in persons without a clinical history of neurological symptoms suggestive of central nervous system demyelination (20). Despite growing research and clinical interest in RIS, its epidemiology remains unclear. Data on its diagnosis in various settings and populations, its natural course, and predictors are sorely needed. Over half of RIS patients were recently shown to develop MS over 10 years of follow-up in the largest international series (29). Whether to treat persons with RIS using MS DMT is debated and currently assessed in clinical trials (NCT02739542, NCT03122652), but clinical follow-up is strongly recommended to initiate treatment early if clinical symptoms arise. In terms of frequency, a systematic review based on autopsy studies and clinical registries of MRI data found a cumulative incidence of RIS of ~0.1% (30). Hospital-based studies estimated a prevalence of 0.05% in the broad age group of 0–90 years (0.15–0.7% in 15–40 years) (31, 32). Another study that collected data from all imaging centers in a region of Sweden, over a year, thus reflecting a population-based catchment area, reported a prevalence of 0.1% among 1,907 individuals aged 0–91 years (33). Because it has been suggested that estimated prevalence rates of an abnormality within the general population cannot serve as a meaningful standard against which to interpret rates of corresponding serious IF in a given sample (4), the need for data in young healthy adults in a research setting is crucial.

### Strengths and Limitations

Strengths of our study include: the large sample size in an understudied age group; high resolution 3T brain MRI; systematic screening of all images by a professor of neuroradiology or MD highly trained in brain imaging studies followed by a second review by a highly trained clinical neuroradiologist (in case of IF discovery), all blinded to the participants' clinical status, in order to have the best accuracy in terms of lesion detection while reducing diagnostic errors; extensive follow-up investigations enabling more accurate characterization of IFs. The management procedure of IFs was optimized through consultations with the ethics advisory board of the i-Share study and additionally reviewed by an independent ethics adviser.

We acknowledge limitations. First, our study sample is not representative of all young adults aged 18–35 years in the Bordeaux area. Only 45% of young adults pursue higher education. Moreover, i-Share participants are not representative of all students, as is the case in all prospective population-based studies, regardless of the sampling method used, with a broader participation of women and most likely a selection bias toward students with an interest in health research. Finally, MRi-Share participants reported significantly better socioeconomic conditions than other i-Share participants in the Bordeaux area, were more often students in the health sector, and older than i-Share participants not taking part in MRi-Share. A selection bias of MRi-Share participants toward students with a history of neurological symptoms cannot be excluded, although compared to other i-Share participants they tended to more often self-rate their health as good or very good and less frequently present a history of migraine. Second, we did not evaluate non-medical consequences of reporting IFs to study participants. However, we carefully designed the IF disclosure process to minimize anxiety, offered psychological support to participants when needed, and made arrangements with medical specialists to reduce the waiting time for follow-up medical visits. The financial impact of IF disclosure was minimized by the fact that the French public national health insurance system carries the main financial burden of medical follow-up. Furthermore, to make up for costs not covered by the aforementioned system, students could access complementary private health insurances covered either by their parents' health insurance plans or through the purchase of their own insurance plan at reduced cost. Third, due to its focus on young adults, MRi-Share lacks participants of middle and older ages; this prevented us from exploring whether the prevalence of IFs differed across the adult lifespan through formal statistical comparisons. Fourth, the protocol we implemented to detect and manage brain IFs should be considered within the specific research context of MRi-Share and might not be well-suited in other settings, e.g., in countries where private healthcare predominates, or for studies based on much larger samples given the resources this would require. Guidelines on the management of IFs are thoroughly needed. The experience described here, complementing prior studies, will be informative for scientific societies or expert groups devising such guidelines in the future.

### What This Study Adds and Future Directions

To our knowledge this is the largest study on IFs in young adults in a research setting and the first that used a standardized protocol, optimized through consultations with ethics advisors, with two independent radiological readings of IFs. It is also the first to report on the management and severity of IFs in young adults. Moreover, although our results remain primarily descriptive, as in most of the literature on incidental findings, the fact that we used the same type of MRI scanner, the same image acquisition protocol, and the same IF definitions as in the UK biobank dataset, allows a qualitative comparison of findings.

Our results provide novel insight into the frequency and severity of precisely defined IFs in young adults, and also shed new light on the nature of these IFs, which appears to differ notably from that in older adults. We found that, with MS, RIS was the most common PSIF observed in young healthy research participants, thereby highlighting the importance of ongoing therapeutic trials on the management of RIS. We also observed that incidentally discovered brain tumors in young adults participating in research appeared to differ histologically from those identified in older adults, although no formal statistical comparisons could be performed.

In the future, our study could be complemented by a more extensive exploration of risk factors associated with IFs and their severity in young adults, requiring much larger samples, and by a formal assessment of differences in prevalence and etiology between age groups. Guidelines on the detection and management of IFs would be highly valuable in order to optimize the way IFs are handled and also reduce inconsistencies between studies reporting them. In analogy with recommendations on the management of incidental genetic findings emerging from sequencing studies in research (34), scientific societies or expert groups could propose a list of actionable brain MRI IFs requiring medical referral, ideally with specific recommendations by age group, considering the different patterns observed across the adult lifespan and age-specific clinical implications.

## Conclusion

Our study provides some guidance on the expected frequency and severity of IFs on brain MRI in young healthy adults participating in research, an understudied group, and shows that the etiological pattern of these IFs is distinct from patterns described in older adults. White matter lesions revealing MS or RIS were the most common potentially serious IFs detected in our study. Altogether, our data may inform IF detection and management protocols in future research studies involving brain MRIs of young adults. Given the growing frequency of brain imaging research, with increasingly large samples and high resolution, our findings also highlight the need for expert guidelines on brain MRI IFs management.

## Data Availability Statement

The data analyzed in this study is subject to the following licenses/restrictions: legal and ethical restrictions prohibit public sharing of the individual raw data related to IFs, because such data contain potentially identifiable and sensitive/confidential information on participants. Requests to access these datasets should be directed to Stéphanie Debette, stephanie.debette@u-bordeaux.fr.

## Ethics Statement

The studies involving human participants were reviewed and approved by the Commission Nationale de l'Informatique et des Libertés (CNIL) (DR-2013-019) (for the i-Share project), the regional Ethics Committee (Comité de Protection des Personnes Sud-Ouest et Outre-Mer III) on 24 August 2015 (CPP2015-A00850-49) (for the MRi-Share protocol). IFs' assessment and management protocol was discussed in detail with the independent ethics advisory board of the i-Share study. The patients/participants provided their written informed consent to participate in this study.

## Author Contributions

SD, BM, and CT joint supervisors. CT conceived and designed the i-Share study (principal investigator), and obtained funding for the i-Share cohort. BM conceived and designed the MRi-Share study (principal investigator). SD was involved in the design and conception of the i-share study (co-investigator) and obtained funding for analyst's time. AS and SD conceptualized and developed the question of the study's described in this manuscript. AS cleaned and prepared the IFs dataset. AS and CB cleaned and prepared the portion of the i-Share dataset used in this work, analyzed and conducted statistical analyses. AS, SM, and SD interpreted the study's results. AS drafted the manuscript (with tables/figures). NB, EM, and BM were involved in MRi-Share data acquisition. AL, AT, EM, and BM contributed to MRi-Share imaging data processing (software development for AL and AT). EM, TT, and BM were responsible for MRi-Share imaging data interpretation including flagging and/or confirmation of IFs. AL, AT, and NB contributed to MRi-Share data management. BB, HL, and SD were involved in clinical data acquisition. EP contributed to i-Share planning and data management. CP and MM contributed to i-Share planning and recruitment. ML contributed to data monitoring. All authors critically revised the manuscript and approved the submitted version.

## Conflict of Interest

SD is currently a guest editor for the Research Topic this work will be submitted to, and has collaborated/is collaborating on research projects or publications with the other two editors of this topic. Outside the submitted work, BB served on the scientific advisory board of Biogen, BMS, Novartis, Roche, Merck, and Jansen, received speaker honoraria from Biogen, BMS, and Roche, served as an editorial board member of Multiple Sclerosis International (2019–2020), received research support from Roche, Biogen, Bayer, Sanofi, and Caridian. The remaining authors declare that the research was conducted in the absence of any commercial or financial relationships that could be construed as a potential conflict of interest.

## Footnotes

^1^i-Share est la plus grande étude scientifique jamais réalisée sur la santé des jeunes [Internet]. Université de Bordeaux. Available online at: https://www.i-share.fr (accessed November 24, 2020).

## Abbreviations

IFs: Incidental Findings
PSIFs: Potentially Serious Incidental Findings
UKB: UK biobank.
