# Supplementary material for: Prevalence, Severity, and Clinical Management of Brain Incidental Findings in Healthy Young Adults: MRi-Share Cross-Sectional Study
Source: Front Neurol. 2021 May 20;12:675244. doi: 10.3389/fneur.2021.675244 (PMC8173138; doi:10.3389/fneur.2021.675244)
Supplement: Supplementary file 1 [file Table_1.docx]

**Supplementary Table 1. Details about acquisition parameters of the five MRI modalities**

| **Modality** | **Voxel (matrix) size** | **Parameters** | **Duration** |
| --- | --- | --- | --- |
| T1-weighted (T1w) | 1.0x1.0x1.0 mm^3^  (192x256x256) | 3D MPRAGE, sagittal, R=2, TR/TE/TI=2,000/2.0/880 ms | 4:54 |
| T2-weighted (T2w) FLAIR | 1.0x1.0x1.0 mm^3^  (192x256x256) | 3D SPACE, sagittal, R=2, TR/TE/TI=5,000/394.0/1800 ms, PF=7/8 | 5:50 |
| Diffusion Weighted imaging (DWI) | 1.75x1.75x1.75 mm^3^  (118x118x84) | 2D axial, MB=3, R=1, TR/TE=3,540/75.0 ms, PF=6/8, fat sat, 100 directions, multishell b=0 s/mm^2^ (8PA+8AP), b=300 s/mm^2^ (8 directions), b=1000 s/mm^2^ (32 directions), b=2000 s/mm^2^ (60 directions) | 9:45 |
| Susceptibility-weighted imaging (SWI) | 0.8x0.8x3.0 mm^3^  (252x288x48) | 2D axial, R=2, TR/TE1=24.0/9.42 ms, PF=7/8 | 2:15 |
| Resting-state functional MRI | 2.4x2.4x2.4 mm^3^  (88x88x66) | 2D axial, EchoPlanar Imaging, MB=6, TR/TE=850/35.0 ms, flip angle=56°, fat sat | 14:58 |
| MPRAGE: magnetization-prepared rapid acquisition with gradient echo, TR: repeti­tion time, TE: echo time, TI: inversion time, FLAIR: Fluid-attenuated inversion recovery imaging, SPACE: sampling perfection with application-optimized contrasts using different flip angle evolutions, R: in-plane acceleration factor, PF: partial Fourier, MB: multiband factor, AP/PA: anterior-posterior/posterior-anterior. | | | |
