# Supplementary material for: Prevalence, Severity, and Clinical Management of Brain Incidental Findings in Healthy Young Adults: MRi-Share Cross-Sectional Study
Source: Front Neurol. 2021 May 20;12:675244. doi: 10.3389/fneur.2021.675244 (PMC8173138; doi:10.3389/fneur.2021.675244)
Supplement: Supplementary file 2 [file Table_2.docx]

**Supplementary Table 2. Proportion of participants with incidental findings (IFs) requiring medical referral by type of final diagnoses**

|  | **Prevalence**  **in study sample** | **Prevalence in participants**  **with IFs initially referred** | **Prevalence in participants**  **with PSIFs^*^ and a final diagnosis available** |
| --- | --- | --- | --- |
| **Final diagnosis for referred IFs or for PSIFs, n1/n2** | **(N = 1,867)** | **(N = 36)** | **(N=19)** |
| **Cysts/ventricular abn., n=9/4** | **0.5% (0.2-0.9%)** | **25.0% (13.6-41.3%)** | **21.1% (8.0-43.9%)** |
| Pineal cyst, n=5/0 | 0.3% (0.09-0.7%) | 13.9% (5.6-29.1%) | – |
| Passive hydrocephalus, n=1/1 | 0.05% (0.0-0.3%) | 2.8% (0.0-15.4%) | 5.3% (0.0-26.5%) |
| Arachnoid cyst, n=3/3 | 0.2% (0.03-0.5%) | 8.3% (2.1-22.6%) | 15.8% (4.7-38.4%) |
| **Vascular anomaly, n=6/4** | **0.3% (0.1-0.7%)** | **16.7% (7.5-32.3%)** | **21.1% (8.0-43.9%)** |
| Unique Cavernoma, n=4/4 | 0.2% (0.06-0.6%) | 11.1% (3.8-25.9%) | 21.1% (8.0-43.9%) |
| DVA, n=2/0 | 0.1% (0.0-0.4%) | 5.6% (0.6-19.1%) | – |
| **Inflammatory WMH, n=5/5** | **0.3% (0.09-0.7%)** | **13.9% (5.6-29.1%)** | **26.3% (11.5-49.1%)** |
| RIS, n=3/3 | 0.2% (0.03-0.5%) | 8.3% (2.1-22.6%) | 15.8% (4.7-38.4%) |
| MS, n=2/2 | 0.1% (0.0-0.4%) | 5.6% (0.6-19.1%) | 10.5% (1.7-32.6%) |
| **Other, neurological, n=5/1** | **0.3% (0.09-0.7%)** | **13.9% (5.6-29.1%)** | **5.3% (0.0-26.5%)** |
| WMH without underlying inflammatory disease, n=3/0 | 0.2% (0.03-0.5%) | 8.3% (2.1-22.6%) | – |
| Undetermined leukoencephalopathy, n=1/0 | 0.05% (0.0-0.3%) | 2.8% (0.0-15.4%) | – |
| Fahr's syndrome, n=1/1 | 0.05% (0.0-0.3%) | 2.8% (0.0-15.4%) | 5.3% (0.0-26.5%) |
| **Tumors, n=4/4** | **0.2% (0.06-0.6%)** | **11.1% (3.8-25.9%)** | **21.1% (8.0-43.9%)** |
| Ganglioglioma, n=1/1 | 0.05% (0.0-0.3%) | 2.8% (0.0-15.4%) | 5.3% (0.0-26.5%) |
| Ependymoma, n=1/1 | 0.05% (0.0-0.3%) | 2.8% (0.0-15.4%) | 5.3% (0.0-26.5%) |
| MVNT, n=2/2 | 0.1% (0.0-0.4%) | 5.6% (0.6-19.1%) | 10.5% (1.7-32.6%) |
| **Other, non-neurological, n=3/0** | **0.2% (0.03-0.5%)** | **8.3% (2.1-22.6%)** | – |
| Hypertrophy lymphoid tissue in cavum, n=1/0 | 0.05% (0.0-0.3%) | 2.8% (0.0-15.4%) | – |
| Bone hemangioma, n=1/0 | 0.05% (0.0-0.3%) | 2.8% (0.0-15.4%) | – |
| Cyst of the maxillary sinus, n=10 | 0.05% (0.0-0.3%) | 2.8% (0.0-15.4%) | – |
| **Cortical malformations, n=3/1** | **0.2% (0.03-0.5%)** | **8.3% (2.1-22.6%)** | **5.3% (0.0-26.5%)** |
| Neuronal migration disorder, n=2/0 | 0.1% (0.0-0.4%) | 5.6% (0.6-19.1%) | – |
| Focal cortical dysplasia, n=1/1 | 0.05% (0.0-0.3%) | 2.8% (0.0-15.4%) | 5.3% (0.0-26.5%) |
| **Unavailable, n=3** | – | – | – |
| PSIFs=Potentially Serious Incidental Findings; abn.= abnormalities; DVA=Developmental venous anomaly; RIS= Radiologically Isolated Syndrome; MS= Multiple Sclerosis; MVNT= Multinodular and Vacuolating Neuronal Tumor;  n1=number of participants classified by type of final diagnoses of interest associated to their referred IFs; n2= number of participants classified by type of final diagnose of interest associated to their PSIFs | | | |
| The sum of all participants classified by type of final diagnoses of interest associated to referred IFs (n=35, sum of all n1) is different than the number of participants requiring medical referral (n=36) or requiring medical referral with a final diagnosis available (n=33) because three participants referred had no final diagnosis available and two participants referred had two different IFs. Prevalences and their 95% confidence intervals are presented. A prevalence of interest is computed as the ratio of the number of participants with referred IFs and the final diagnosis of interest (n1) or the number of participants with PSIFs and the diagnosis of interest (n2) over the size of the sample mentioned in the relevant column, e.g., vascular anomaly was observed in 6 participants with IFs referred among the 1,867 MRi-Share participants (prevalence of 0.5% in the study sample) and in 4 out of the 19 participants with PSIFs followed-up for final diagnosis (prevalence of 21.1%) | | | |
| ^*^ IFs referred that were retrospectively identified according to the list of PSIFs developed by the UK biobank, or its definition of PSIFs, with clinical, imaging and/or biological examinations (follow-up procedures) to provide final diagnosis | | | |
